# Testing association and maternally mediated genetic effects with the principal component analysis in case-parents studies

**DOI:** 10.1186/s12863-016-0336-y

**Published:** 2016-01-19

**Authors:** Yumei Li, Yang Xiang

**Affiliations:** School of Mathematics and Computational Science, Huaihua University, Huaihua, Hunan 418008 P. R. China

**Keywords:** Association analysis, Maternally mediated genetic effects, Case-parent triads, Principal component analysis

## Abstract

**Background:**

Major advances in genotyping technology have generated high-density maps of single nucleotide polymorphism (SNP) markers that provide an unprecedented opportunity to identify genes underlying complex traits. Several family-based statistical methods showing robust population stratification have been developed to test the association between multiple markers and disease-susceptibility genes. Only a few methods focus on testing for maternally mediated genetic effects, which is a critical risk for birth defects. The present study focuses on testing for association and maternally mediated genetic effects with family-based methods.

**Results:**

In the present study, we proposed a new method, max_*PC* integrating principal component analysis, to test association or maternally mediated genetic effects with case-parent data. The proposed method only uses the genotypes of case-parents triads and accommodates missing SNP data. Our results demonstrated that this method is powerful to test association or maternally mediated genetic effects and attractive because it provides a tool for testing the null hypothesis of no association and no maternally mediated genetic effects. Simulations with the permutation procedure as well as an application in the Crohn’s disease study showed that the type I error rates of the proposed statistic were nominal with slightly higher power as compared to those of the max_Z^2^ test.

**Conclusions:**

We conclude that the max_*PC* is a good approach to test association or maternally mediated genetic effects.

## Background

The rapid advancement of genotyping technologies and the availability of enormous quantities of genotype or haplotype data provide an unprecedented opportunity for identifying genes involved in susceptibility to complex diseases. The utilization of information from individual markers as well as linkage disequilibrium (LD) structure between the markers has resulted in the use of haplotype-based association studies to determine the relationship between complex traits and a series of possibly linked markers. However, haplotype-based methods are challenging as the large number of distinct haplotypes will in turn produce a large number of degrees of freedom. In addition, some haplotype-based methods need the estimation of haplotype phases when haplotypes are not directly observed. A potential strategy to avoid the estimation of haplotype phases and frequencies is to develop genotype-based statistical approaches that directly use genotype data.

Population-based case–control studies and family-based studies are two common strategies for association analysis. Compared to case–control studies, family-based studies are more attractive due to their robustness to population stratification. Several genotype-based methods with case-parent data have been proposed for association test. McIntyre et al. [1] proposed a max_TDT test, in which the usual TDT statistic for each locus was calculated and the maximum was utilized as the test statistic. Using the principal components (PC) of a variance-covariance matrix of difference vectors that were calculated by comparing the genotypes of affected offspring with their corresponding “complements”, which are defined to be the parental genotypes that were not present in the affected offspring, Lee WC [2] proposed the adaptive principal component test, *PCT*_*L*_^2^. Based on the same difference vector, Fan et al. [3] proposed a paired Hotelling’s T^2^ test, while Shi et al. [4] developed the max_Z^2^ test. The max_Z^2^ test is superior to other methods as it is able to study the maternally mediated genetic effects while only using the genotypes of affected individuals and their parents. It could also accommodate missing SNP data. Maternally mediated genetic effects arise when the phenotype of an offspring is influenced by maternal alleles via the intrauterine environment [5]. This effect can be detected by using the father’s genotype as a matched control for the mother’s under an assumption of mating symmetry [6]. The maternal genome influences the risk for specific diseases in the offspring, suggesting maternal genes play an independent role in the etiology of birth defects [5, 6]. So incorporating maternally mediated genetic effects into an association analysis might be particularly useful in studying diseases that happened during fetal life.

In this report, we proposed a new statistic, max_*PC*, to test association and maternally mediated genetic effects with case-parent data. Here, the null hypothesis is that neither association nor maternally mediated genetic effects exist. The max_*PC* test has three characteristics: (1) only using the genotypes of case-parent triads while accommodating missing SNP data, (2) utilizing the principal component analysis, and (3) evaluating the association and the maternally mediated genetic effects at the same time. Simulations analysis using a permutation procedure and an application in Crohn’s disease study indicated that this max_PC statistic has good performance.

## Results

### Simulation setting

To assess the performance of the statistic, max_*PC*, we performed a simulation study using a wide range of parameters. The simulations were implemented as described by Lee WC [2]. A candidate region of 100 kb in size was considered, where 20 dense marker loci were uniformly distributed in a homogeneous population. The marker frequencies were randomly determined, with values ranging from 0.1–0.9, and the disequilibrium coefficients between two adjacent markers were uniformly generated between −0.9 and 0.9 [2, 7]. Assumption was made that a biallelic disease susceptibility gene with alleles *D* and *d* was uniformly located within the candidate region. The frequency of disease allele *D* was set to be 0.1. Let R_1_ denote the relative risk with one copy of the disease allele D and R_2_ denote the relative risk with two copies compared with no copies. We considered a simple mutational process for the disease allele *D* where the allele *D* appears in an individual with the “ancestral haplotype” and it broke up because of meiotic recombination. We used the first-order Markov model [8] to generate the markers on the ancestral haplotype and then generate the haplotypes in the present-day population.

For the null hypothesis that neither association nor maternally mediated genetic effects exist, we let R_1_ = R_2_ = 1. For the alternative hypothesis that there exists association or maternally mediated genetic effects, we considered the following three scenarios. First, there exists association where the state of disease phenotype of the offspring was determined by the offspring genotype; the second, there exists maternally mediated genetic effects where the state of disease phenotype of the offspring was determined by the mother genotype; and third, there exist both association and maternally mediated genetic effects where the state of disease phenotype of the offspring was determined by the genotypes of both mother and offspring. For the third scenario, we considered a simple model in which the relative risk was the product of the relative risks of the offspring and the mother. For the above three scenarios, we let the baseline disease risk (the penetrance of genotype *dd*) be 0.01, and R_1_ = R_2_ = 2 and R_1_ = 1, R_2_ = 2, which represented a dominant and a recessive model, respectively. The number of case-parent triads, *N*, was chosen as 100, 200, or 400. We assumed that each individual had an available marker that could be utilized as a genotype in the analysis. Upon simulation of the familial data, we first calculated the value of max_*PC,* and then recalculated it by using the permutation procedure 1,000 times. The $\widehat{p}$ value was the proportion of permutation-based statistics that were larger than the data-based statistic. We performed this data simulation with 10,000 replicates. For a given significance level *α*, the power/type I error rate is then estimated as the proportion of rejecting the null hypothesis when $\widehat{p}\le \alpha$.

We also investigated the performance of max_*PC* in the presence of population admixture. We assumed that there were two subpopulations that differed in disease allele frequency and baseline disease risk. Disease allele *D* was introduced 1,000 and 500 generations ago through two different ancestral haplotypes in the two subpopulations, respectively. The frequencies of the *D* allele were set as 0.1 and 0.2, and the baseline disease risks were set as 0.01 and 0.05 in the first and second subpopulation, respectively. We used the previously described approach to generate the markers and haplotypes in each subpopulation and let the two subpopulations admix at the last generation, with 30 percent for the first subpopulation and 70 percent for the second subpopulation.

### Assessment of the performance of the max_*PC* statistic

As shown in Table 1, the type I error rates are around the nominal value, indicating that the type I error rates are validated in both homogeneous and admixture populations. The powers under three scenarios are exhibited in Tables 2 and 3. The power of max_Z^2^ was also investigated so that we could compare the performance of max_*PC* with that of max_Z^2^. It should be noted that max_Z^2^ uses the difference vector 2*C* − *F* − *M* and *M* − *F* under the first scenario and the second scenario, respectively. However, we perform two tests under the third scenario: one uses the difference vector 2*C* − *F* − *M* to calculate max_Z^2^ for testing association and the other uses the difference vector *M* − *F* to calculate max_Z^2^ for testing maternally genetic effects. It is observed from Table 2 that, in homogeneous population, the power of max_*PC* for testing association and maternally mediated genetic effects is highest among three scenarios. The powers for testing association and maternally mediated genetic effects reach 80 % and 90 %, respectively, when the sample size is 200 or 400. The power of max_*PC* under the recessive model is slightly lower than those under the dominant mode. The second to the fifth columns of Table 2 show that the powers of max_*PC* are similar to those of max_Z^2^. Under the third scenario of testing association and maternally mediated genetic effects at the same time, the powers of max_*PC* are slightly higher than those of max_Z^2^ with the difference vector 2*C* − *F* − *M* and those of max_Z^2^ with *M* − *F*. Table 3 presents the powers in admixture population. It can be seen that the results are similar to those observed in homogeneous population.

**Table 1:** Estimated type I error rates of the statistic, max_*PC*, for 10,000 simulations

	Estimated type I error rates					
	Homogeneous population			Admixture population		
Sample size	*α* = 0.05	*α* = 0.01	*α* = 0.001	*α* = 0.05	*α* = 0.01	*α* = 0.001
100	0.0486	0.0106	0.0012	0.0490	0.0095	0.0010
200	0.0502	0.0092	0.0009	0.0487	0.0105	0.0009
400	0.0489	0.0089	0.0018	0.0521	0.0110	0.0013

**Table 2:** The powers of max_*PC* and max_Z^2^ for various tests based on simulations with global significance at *p* ≤ *α* (0.05) in a homogeneous population

Test	Association		Maternally mediated genetic effects		Association and maternally mediated genetic effects		
*N* = 100	max_*PC*	max_Z^2^	max_*PC*	max_Z^2^	max_*PC*	max_Z^2 a^	max_Z^2 b^
R_1_ = R_2_ = 2	0.616	0.607	0.588	0.586	0.815	0.800	0.584
R_1_ = 1, R_2_ = 2	0.600	0.582	0.579	0.581	0.808	0.787	0.582
*N* = 200							
R_1_ = R_2_ = 2	0.819	0.820	0.725	0.711	0.917	0.902	0.713
R_1_ = 1, R_2_ = 2	0.803	0.810	0.702	0.704	0.911	0.898	0.702
*N* = 400							
R_1_ = R_2_ = 2	0.908	0.900	0.819	0.821	0.952	0.937	0.822
R_1_ = 1, R_2_ = 2	0.875	0.874	0.807	0.809	0.926	0.915	0.810

^a^The max_Z^2^ using 2*C* − *F* − *M*

^b^The max_Z^2^ using *M* − *F*

**Table 3:** The power of max_*PC* for various tests based on simulations with global significance at *p* ≤ *α* (0.05) in an admixture population

Test	Association		Maternally mediated genetic effects		Association and maternally mediated genetic effects		
*N* = 100	max_*PC*	max_Z^2^	max_*PC*	max_Z^2^	max_*PC*	max_Z^2 a^	max_Z^2 b^
R_1_ = R_2_ = 2	0.602	0.600	0.564	0.561	0.803	0.787	0.562
R_1_ = 1, R_2_ = 2	0.587	0.576	0.551	0.548	0.788	0.769	0.547
*N* = 200							
R_1_ = R_2_ = 2	0.805	0.806	0.709	0.711	0.855	0.850	0.712
R_1_ = 1, R_2_ = 2	0.779	0.783	0.682	0.683	0.834	0.826	0.684
*N* = 400							
R_1_ = R_2_ = 2	0.878	0.879	0.800	0.798	0.882	0.875	0.796
R_1_ = 1, R_2_ = 2	0.864	0.867	0.776	0.777	0.879	0.870	0.769

^a^The max_Z^2^ using 2*C* − *F* − *M*

^b^The max_Z^2^ using *M* − *F*

### A real example

**Fig. 1:**
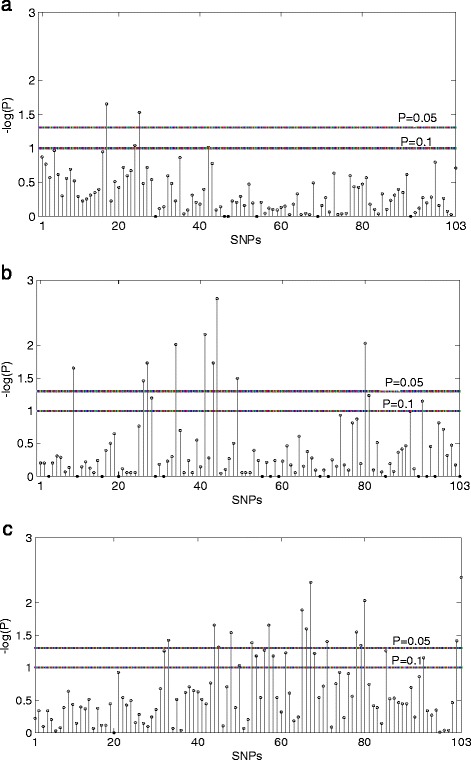
Crohn’s disease study. Result of testing association using the max_Z^2^ with *M* − *F* (**a**), testing maternal effects using the max_Z^2^ with 2*C* − *F* − *M* (**b**), and testing association or maternal effects using the max_*PC* (**c**). The *Y*-axis shows –log10(p) at individual SNPs; the *X*-axis shows SNPs

To evaluate the performance of the max_*PC* on more realistic scenarios, we applied our method to a real data set of Crohn’s disease. Crohn’s disease is one of the two major forms of inflammatory bowel diseases [9, 10]. Rioux et al. [10, 11] detected a candidate region containing a genetic risk factor for Crohn disease on human chromosome 5q31with 139 case-parents trios. We applied the max_*PC* and the max_Z^2^ to the publically available subset of 129 trios genotyped at 103 common SNPs. We assessed *P* value using the permutation procedure 10,000 times. The *P* value using the max_*PC* is 3.62 × 10^−4^, indicating that this region is associated with Crohn’s disease or there is a possible maternal effects. When the max_Z^2^ with 2*C* − *F* − *M* and *M* − *F* was used, the *P* values are 3.78 × 10^−4^ and 4.05 × 10^−3^, respectively, indicating that there may exist association and maternal effects. In order to further compare the two methods, we also considered risk-haplotype-tagging alleles which are highly predictive of disease susceptibility as descripted by Shi et al. [4]. According to Shi et al. [4], we consider the locus with *P* value being smaller than a preset threshold to be related to risk and designate the corresponding overtransmitted allele as a risk-haplotype-tagging allele. It can be seen from Fig. 1 that the number of risk-haplotype-tagging alleles identified using the max_*PC* (Fig. 1c) is larger than that using the max_Z^2^ (Fig. 1a and b).

## Discussion

Based on the principal component analysis, we proposed a statistic max_*PC* to test association and maternally mediated genetic effects with multiple markers. Similar to the max_Z^2^ test, the max_*PC* test only uses the genotypes of case-parent triads and accommodates missing SNP data. Notably, at least two characteristics of the max_*PC* test are totally different from those of the max_Z^2^ test: (1) the max_*PC* test uses the principal component analysis, and (2) the max_*PC* can be used to test association and maternally mediated genetic effects at the same time, whereas two tests with max_Z^2^ should be performed for this case. The performance of max_*PC* was assessed by simulations analysis as well as the application in Crohn’s disease study.

In practice, when multiple markers are studied, individuals may have incomplete information of individual marker data. Our method calculates the *PC* value for each locus, indicating that it is capable of handling missing SNP data. Moreover, our approach is not biased toward admixture population and Hardy-Weinberg equilibrium was not essential when using our method. The null hypothesis for the max_*PC* test is that there is no association and no maternally mediated genetic effects, whereas the alternative hypothesis is that there is an association or maternally mediated genetic effects. One attractive feature of max_*PC* is that it is able to test association and maternally mediated genetic effects exist at the same time. Also, it provides a method for testing the null hypothesis of no association and no maternally mediated genetic effects. When the null hypothesis is rejected we will use max_Z^2^ with the difference vector, 2*C* − *F* − *M*, for testing association, and use max_Z^2^ with the difference vector, *M* − *F*, for testing maternally mediated genetic effects. In this way, we will be able to determine whether only maternally mediated genetic effects or only association exists. It is worth noting that testing for maternally mediated genetic effects rely on the assumption of mating symmetry when max_*PC* was used according to *M* − *F*. However, when an offspring effect is present, parent-of-origin effects can also cause the effect of symmetry [4]. In this case, it is not easy to distinguish maternally mediated genetic effects from parent-of-origin effects. Further studies to carefully evaluate the possible parent-of-origin effects are thus warranted.

## Conclusions

We have proposed a statistical approach for detecting association or maternally mediated genetic effects with case-parent data. The approach is powerful to test association and the maternally mediated genetic effects at the same time. It also provides a valid tool for testing no association and no maternally mediated genetic effects. The proposed method for association analysis incorporating maternally mediated genetic effects is particularly useful in studying diseases that happened during fetal life.

## Methods

In this study, all datasets were publically available and no research requiring ethics approval was conducted.

We consider a biallelic marker with alleles “A” and “a”. Assume that case-parent triads are sampled with the genotypes known for each member of the triads. Let *M*, *F*, and *C* be the number of copies of allele “A” carried by the mother, father, and affected offspring, respectively. 2*C* − *F* − *M* is the paired differences in genotypes between the affected offspring and the complement, which carries the non-transmitted genotypes in the case-parent data. *M* − *F* is the SNP-count difference between the mother and the father, which can be used to measure the maternally mediated genetic effects with the assumption of mating symmetry. Let *X* = 2*C* − *F* − *M* and *Y* = *M* − *F. X* = 0 when there is no association, and *Y* = 0 when there is no maternally mediated genetic effects. Define a two-dimensional random variable *Z* = (*X*, *Y*)^*T*^. Under the null hypothesis of no association and no maternally mediated genetic effects, *Z* = 0. We assume that there are *n* case-parent triads. Let *S* be the sample covariance matrix of *Z* for the observed case-parent trios data, *Z*_*i*_ = (*X*_*i*_, *Y*_*i*_)^*T*^ (*i* = 1, ⋯, *n*). Denote the two sample eigenvalue-eigenvector pairs for *S* by $\left({\widehat{\lambda}}_1,{\widehat{e}}_1\right)$, $\left({\widehat{\lambda}}_2,{\widehat{e}}_2\right)$, where ${\widehat{\lambda}}_1\ge {\widehat{\lambda}}_2\ge 0$. Then, the *k*th (*k* = 1, 2) sample principal component is *ζ*_*k*_ = *ê*_*k*_^*T*^*Z*, with variance of $Var\left({\zeta}_k\right)={\widehat{\lambda}}_k$. Note that the two sample principal components *ζ*_1_, *ζ*_2_ are uncorrelated, i.e., *Cov*(*ζ*_1_, *ζ*_2_) = 0. The *k*th sample principal component for the *i*th case-parent trio is *ζ*_*ik*_ = *ê*_*k*_^*T*^*Z*_*i*_ (*k* = 1, 2; *i* = 1, ⋯, *n*). Let ${\overline{\zeta}}_k=\frac{1}{n}\underset{i=1}{\overset{n}{\varSigma }}{\zeta}_{ik}$. Here, $E\left({\overline{\zeta}}_k\right)=E\left({\zeta}_k\right)$, $Var\left({\overline{\zeta}}_k\right)=\frac{1}{n}{\widehat{\lambda}}_k$, and $Cov\left({\overline{\zeta}}_1,{\overline{\zeta}}_2\right)=0$ (*k* = 1, 2). Under the null hypothesis of no association and no maternally mediated genetic effects, $E\left({\overline{\zeta}}_k\right)=0$ for *k* = 1, 2. Define a statistic, denoted by *PC*, as follow:

$$ PC=\frac{n{\left(\underset{k=1}{\overset{2}{\varSigma }}{\overline{\zeta}}_k\right)}^2}{\underset{k=1}{\overset{2}{\varSigma }}{\widehat{\lambda}}_k} $$

The statistic *PC* is asymptotically a central *χ*_(1)_^2^ distribution under the null hypothesis of no association and no maternally mediated genetic effects.

Now, we consider *q* biallelic markers each with alleles “A” and “a”. The markers are indexed by *l* (*l* = 1, ⋯, *q*). Let *PC*^(*l*)^ be the statistic for marker *l*. Our test statistic, here, denoted as max_*PC*, is the maximum of the *PC*^(*l*)^ across all *q* marker loci, i.e., $\max \hbox{-} PC=\underset{l}{ \max}\left\{P{C}^{(l)}\right\}$. It can be seen that this max_*PC* approach is able to accommodate missing individual marker genotypes and does not require any haplotype information. Here, the statistical significance is assessed by a permutation procedure. We first calculate the data-based statistic. Then we permute the “case” and “complement” labels with equal probability and recalculate the statistic. The estimated *P* value (denoted by $\widehat{p}$) is then the proportion of permutation-based statistics that are larger than the data-based statistic.

### Availability of supporting data

The data set used in this article is shown in Tables 1, 2 and 3. Data of Crohn’s disease are available from http://www.broadinstitute.org/archive/humgen/IBD5/raw_data.txt and http://www.broadinstitute.org/archive/humgen/IBD5/haplodata.html.
